# Carotenoids from Cyanobacteria: Biotechnological Potential and Optimization Strategies

**DOI:** 10.3390/biom11050735

**Published:** 2021-05-15

**Authors:** Fernando Pagels, Vitor Vasconcelos, Ana Catarina Guedes

**Affiliations:** 1 CIIMAR—Interdisciplinary Centre of Marine and Environmental Research, University of Porto, Novo Edifício do Terminal de Cruzeiros de Leixões, Avenida General Norton de Matos, s/n, 4450-208 Matosinhos, Portugal; fernandopagels@gmail.com (F.P.); vmvascon@fc.up.pt (V.V.); 2 FCUP—Faculty of Science, University of Porto, Rua do Campo Alegre, s/n, 4169-007 Porto, Portugal

**Keywords:** xanthophylls, carotenes, orange carotenoid protein, bioactive potential, production, extraction, purification

## Abstract

Carotenoids are tetraterpenoids molecules present in all photosynthetic organisms, responsible for better light-harvesting and energy dissipation in photosynthesis. In cyanobacteria, the biosynthetic pathway of carotenoids is well described, and apart from the more common compounds (e.g., β-carotene, zeaxanthin, and echinenone), specific carotenoids can also be found, such as myxoxanthophyll. Moreover, cyanobacteria have a protein complex called orange carotenoid protein (OCP) as a mechanism of photoprotection. Although cyanobacteria are not the organism of choice for the industrial production of carotenoids, the optimisation of their production and the evaluation of their bioactive capacity demonstrate that these organisms may indeed be a potential candidate for future pigment production in a more environmentally friendly and sustainable approach of biorefinery. Carotenoids-rich extracts are described as antioxidant, anti-inflammatory, and anti-tumoral agents and are proposed for feed and cosmetical industries. Thus, several strategies for the optimisation of a cyanobacteria-based bioprocess for the obtention of pigments were described. This review aims to give an overview of carotenoids from cyanobacteria not only in terms of their chemistry but also in terms of their biotechnological applicability and the advances and the challenges in the production of such compounds.

## 1. Introduction

Cyanobacteria, as photosynthetic organisms, have a light-harvesting complex for the absorption of light energy for photosynthesis. This harvesting complex is composed of pigments that can be divided into three chemical groups: chlorophylls, phycobiliproteins, and carotenoids [1]. Such compounds are organized in the thylakoid membrane, in parallel to the cell membrane. Specifically, cyanobacteria have a phycobilisome (containing phycobiliproteins) and orange carotenoid proteins (OCP; containing hydroxyechinenone) outside the membrane, while chlorophyll and most carotenoids are located inside the photosystem, in a transmembrane protein complex [2,3].

In cyanobacteria, the main pigments for light absorption are phycobiliproteins, while carotenoids have a huge impact on the non-photochemical quenching (NPQ) as protection agents against saturating light and quencher of reactive oxygen species (ROS) [1].

When it comes to carotenoids, these terpenoids pigments are considered essential for the survival of all photosynthetic organisms and are transversal through these organisms. A large number of pigments are described as carotenoids (ca. 600) and can be divided into two major classes, carotenes, such as α- and β-carotene, and xanthophylls (oxygenised derivatives of carotenes) such as zeaxanthin and echinenone [4,5]. Apart from their biological role, carotenoids attract great interest from the industry due to their bioactive potential as antioxidant, anti-inflammatory, and anti-tumoral, among others [4,5,6].

The market value of carotenoids surpassed $1.5 billion (USD) from 2016 to 2019, β-carotene, astaxanthin, and lutein being responsible for 60% of this market [7,8]. The bioprocess of these carotenoids is well established in the microalgae *Dunaliella salina* (β-carotene) and *Haematococcus pluvialis* (astaxanthin) and in the vascular plant marigold *Tagetes erecta* (lutein).

Despite the potential of cyanobacteria as a producer of pigments, the only large-scale market application of these organisms is related to the phycocyanin production from *Arthrospira platensis*. The reason is that the content of carotenoids in cyanobacteria is significantly lower than microalgae or vascular plants [5].

On the other hand, strategies for the use of cyanobacterial carotenoids increased in recent years, translating into a possible *light* for a future *bloom* of cyanobacteria in this market. These strategies go into three main aspects of the bioprocess: (i) in the valorisation of cyanobacterial carotenoids´ extracts by exploiting bioactive potential and unique applicability into nutraceuticals, cosmetics, feed, among others; (ii) in the increase of carotenoids content in cyanobacteria by optimising growth conditions, by using pathway triggers/stress stimuli, or by increasing gene expression through genetic engineering; (iii) in the efficient use of the biomass by efficient extraction and by the use of biorefineries, becoming a co-product of phycobiliproteins production.

This paper aims to review the different kinds of cyanobacterial carotenoids as well as the most relevant characteristics for biotechnological application, with a specific focus on their bioactivities. The optimisation of their production, extraction, and purification strategies is also emphasized. Finally, economic considerations and future perspectives in the field are briefly discussed.

## 2. Chemistry

Carotenoids are tetraterpenoids molecules, i.e., most carotenoids are composed of a C40 hydrocarbon chain containing eight isoprenoids and a series of double-bound conjugations. They can subdivide into carotenes, which are either linear or cyclized molecules with one or two rings at their extremes, lacking oxygen atoms; and xanthophylls, which are oxygenated derivatives of carotenes. Moreover, modifications to carotenes can form glycosylated carotenoids such as myxoxanthophyll or even shortened chain carotenoids (apocarotenoids) [5].

**Figure 1:**
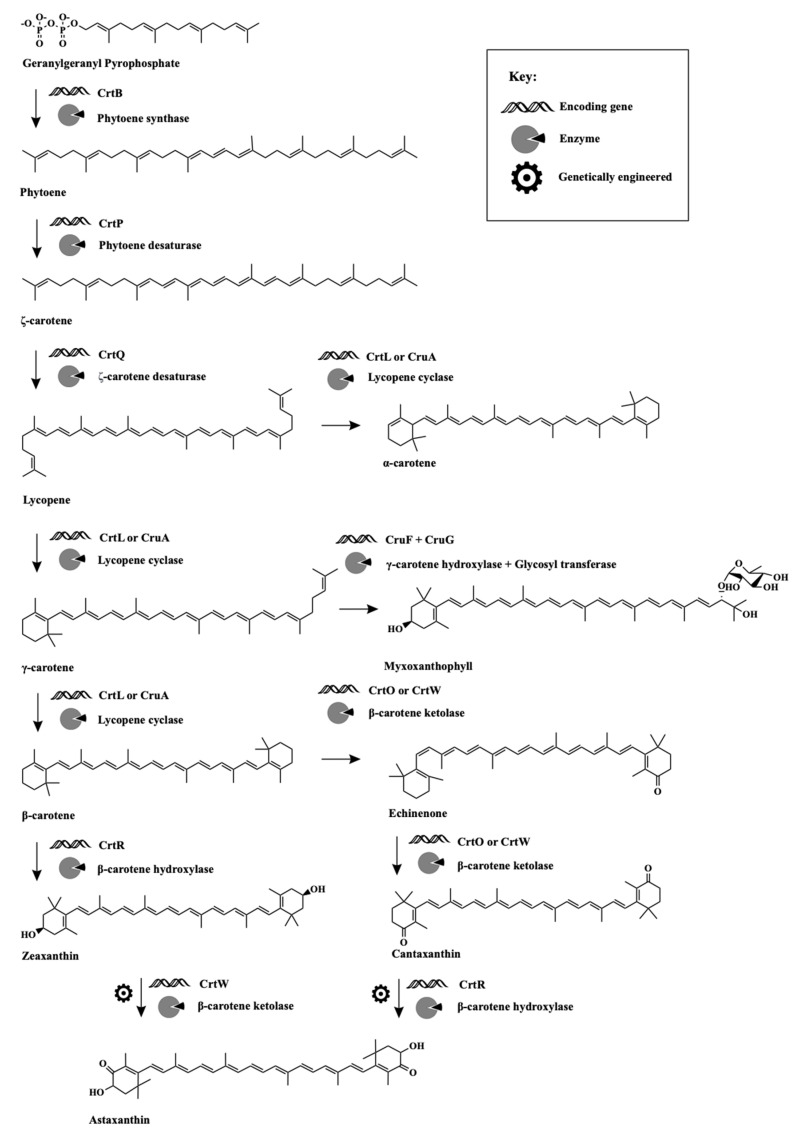
Biosynthetic pathway of carotenoids in cyanobacteria.

Cyanobacteria are able to synthesize a wide variety of terpenoids due to a complex but well-described biosynthetic pathway known as carotenogenesis (Figure 1). The production of these compounds originates from geranylgeranyl pyrophosphate precursor [9,10]. A series of genes encode synthases, desaturases, cyclases, and hydroxylases responsible for the synthesis of carotenoids. Liang et al. [9] evaluated the presence of the described genes through several cyanobacterial species and found that most of the genes are transversal, although specific genes can be found as a replacement or as redundant to other genes.

The biosynthetic pathway starts with two molecules of geranylgeranyl pyrophosphate condensed into phytoene through phytoene synthase (CrtB); from that, a phytoene desaturase can convert phytoene to ζ-carotene (CrtP), and then a carotene desaturase (CrtQ) converts it to lycopene. A few genera such as *Anabaena* and *Nostoc* can convert phytoene directly to lycopene using a phytoene desaturase (CrtI), although they also produce the regular phytoene desaturase (CrtP) [9]. From lycopene, the major primary carotenoids are formed, α- and β-carotene. α-carotene is formed in a direct conversion by lycopene cyclase (CrtL or CruA), while β-carotene is derived from a γ-carotene in a two-step process performed by a lycopene cyclase (CrtL or CruA) [9,10]. From the three carotenoids, several xanthophylls can be formed as follows.

From γ-carotene, through a hydroxylation by γ-carotene hydroxylase (CruF) and the addition of a glycoside group by a glycosyltransferase (CruG), a myxoxanthophyll molecule is formed. Myxoxanthophyll is a yellow glycoside terpenoid exclusive to cyanobacteria, and its production is required for cell wall structure and thylakoid organization [11].

From α-carotene, lutein can be formed by a hydroxylase (CrtR) [12].

From β-carotene, a ketolase (CrtO or CrtW) can convert β-carotene to an echinenone, and a hydroxylase (CrtR) can convert to zeaxanthin. Furthermore, from echinenone, a ketolase (CrtO or CrtW) can form canthaxanthin, and a hydroxylase (CrtR) can form hydroxyechinenone, the main ketocarotenoid in the OCP. From zeaxanthin, both antheraxanthin and violaxanthin can be formed through a revertible activity of epoxidase and de-epoxidase in a process called the violaxanthin cycle. From zeaxanthin, it is also possible to form nostoxanthin by a hydroxylase (CrtG), while from violaxanthin, it is possible to form neoxanthin by a neoxanthin synthase (NSY) [9,10]. Moreover, astaxanthin can be produced using engineered cyanobacteria by the inclusion of a CrtR or a CrtW gene. Astaxanthin can be derived from canthaxanthin and formed by a hydroxylase (CrtR) and derived from zeaxanthin, formed then by a ketolase (CrtW) [13].

Although carotenogenesis genes are conserved in most photosynthetic organisms, the expression and the consequent carotenoid production can be limited to some groups of cyanobacteria [14] and are specifically manipulated by growth conditions [6]. In a biotechnological approach, it is fundamental to take advantage of such regulations to increase the production of specific or total carotenoids.

## 3. Orange Carotenoid Protein (OCP)

**Figure 2:**
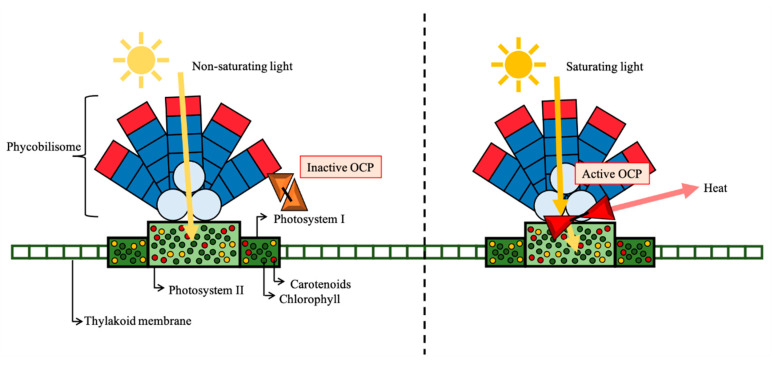
Orange carotenoid protein (OCP) energy dissipation under saturation light.

The cyanobacteria light-harvesting occurs mainly through the phycobilisome, a protein component of the extramembrane antenna pigment (phycobiliproteins), which transfers the energy to the photosystem core [15]. On the other hand, the main function of carotenoids in cyanobacteria is energy dissipation and protection against oxidative damage. The NPQ is responsible to reduce excessive light energy to reach the photosystem core. In cyanobacteria, carotenoids can be present in the reaction centre, together with the chlorophyll molecules, or in a protein complex containing a single molecule of carotenoid, which is a key component for the photoprotection—the OCP [16]. The OCP is a water-soluble protein (35 kDa) containing a single molecule of hydroxyechinenone present in an inactive form (orange) that is triggered by blue-green light and converted to an active form (red) (Figure 2). The OCP, although fundamental for cell protection, usually represents only 1% of total carotenoids [2].

The OCP is structured by two domains joined by a flexible linker. The first, exclusive to cyanobacteria, is an all-helical N-terminal; and the second, found across all kingdoms, is an α/β-fold C-terminal domain [17]. The OCP is encoded by an slr1963 gene [18] constitutively expressed, although stress conditions such as high lighting or salt stress can induce a temporary acclimation and an increase of the transcription of the gene [17].

In summary (as observed in Figure 2), the OCP only attaches to the phycobilisome in its red form (activated). In darkness or low-light conditions, the phycobilisome can absorb and transfer all the energy to the photosystem. In saturation light (strong blue-green or white), OCP changes its conformation to an active form and attaches the phycobilisome for energy dissipation, allowing the non-saturating absorption by the photosystem. Besides phycobiliproteins and OCP, other proteins are involved in the process, such as the fluorescence recovery protein (FRP), responsible for inactivating OCP after the NPQ process [17,19].

## 4. Applications of Carotenoids from Cyanobacteria

Carotenoids overall are widely described as bioactive compounds, such as antioxidants, anti-inflammatory, anti-tumoral, and antimicrobial [6], that can be used in animal feed [4] as a colour enhancer and in cosmetical application as antioxidant and anti-ageing components [20]. However, most carotenoids in the industry come from microalgae and plants, and, as consequence, the studies regarding cyanobacteria carotenoids and their activities are limited [6]. Regarding the bioactive screening of pigments, research is usually performed using targeted extracts with organic solvents, such as ethyl acetate, methanol, ethanol, and acetone. Table 1 summarizes the described application of carotenoids from cyanobacteria.

**Table 1:** Potential of carotenoids from cyanobacteria for biotechnological applications.

Application	Product	Main Identified Carotenoids	Source	Assay	Reference
Anti-inflammatory	Acetonic extract	β-carotene and echinenone	*Nodosilinea* (*Leptolyngbya*) *antarctica*	LPS-induced macrophages (RAW 264.7)	[21]
Antioxidant	Acetone extract after water extraction	Zeaxanthin and β-carotene	*Cyanobium* sp.	ABTS^•+^ and ^•^NO	[22]
	Acetonic extract	β-carotene and echinenone	*Arthrospira platensis*	DPPH^•^ and ABTS^•+^	[23]
	Ethyl acetate extract	β-carotene	*Trichodesmium* sp.	FRAP	[24]
	Methanolic extract	Myxoxanthophyll, zeaxanthin, canthaxanthin and α- and β-carotenes	*Lyngbya* sp.	DPPH^•^	[25]
	Ethyl acetate/Methanol extract	Zeaxanthin, myxoxanthophyll, β-carotene, echinenone and β-cryptoxanthin	*Arthrospira platensis* mixed with *Dunaliella salina*	DMBA-induced tumour in hamster	[26]
Antiurolithiasis	Methanol extract	Myxoxanthophyll, zeaxanthin, canthaxanthin and 〈- and β-carotenes	*Pseudanabaena* sp., *Spirulina* sp. and *Lyngbya* sp.	Calcium oxalate crystallization	[25]
Colour Enhancer (Feed)	Raw biomass	Zeaxanthin, β-carotene and myxoxanthophyll	*Arthrospira platensis*	In vivo fish and poultry assays	[27,28,29,30]
Hyaluronidase inhibitor	Ethanol 70% extract	Zeaxanthin, lutein, canthaxanthin and echinenone	*Cyanobium* sp. and *Tychonema* sp.	Hyaluronidase in vitro assay	[31]

ABTS—2,2′-Azino-bis(3-ethylbenzothiazoline-6-sulfonic acid) diammonium salt; DMBA—7,12-dimethylbenzanthracene; DPPH—2,2-diphenyl-1-picrylhydrazyl; FRAP—ferric antioxidant power; LPS—lipopolysaccharide; NO—nitric oxide.

In terms of antioxidant capacity, Kelman et al. [24] screened extracts from *Trichodesmium* sp., *Anabaena flos-aquae*, *Cyanothece* sp., *Prochlorothrix hollandica*, and *Synechococcus* sp. The extraction was performed using ethyl acetate. The highest antioxidant capacity was found in *Trichodesmium* sp., a bloom-forming marine cyanobacterium and a bioassay-guided fractionation identified β-carotene and retinyl palmitate as main antioxidant compounds.

In another case, methanolic pigment-rich extracts from *Pseudanabaena* sp., *Spirulina* sp. and *Lyngbya* sp. were also suggested as antiurolithiasis (prevention against kidney stone disease) in in vitro assays by Paliwal et al. [25]. Paliwal et al. [25] also evaluated antioxidant capacity, in which *Lyngbya* sp. methanolic extract containing myxoxanthophyll, zeaxanthin, canthaxanthin, and α- and β-carotenes, was the one with the highest IC_50_ for DPPH^•^ scavenging assay (59.56 mg.mg_DPPH_^−1^).

Patias et al. [23] evaluated the carotenoid composition and the antioxidant capacity of *Aphanothece microscopica* lipophilic extract (ethyl acetate and methanol). The extract contained a substantial amount of total carotenoid (1 mg.mL^−1^) with 14 identified carotenoids (major carotenoids were β-carotene and echinenone) and had an antioxidant capacity relative to 7.3 μM of α-tocopherol.

Moreover, Park et al. [32] evaluated the carotenoid content and the antioxidant capacity in *A. platensis*. The carotenoid-targeted extract was performed using acetone. Results showed that the biomass contained 4.4 mg.g^−1^ of carotenoids, with major carotenoids being β-carotene and zeaxanthin. Moreover, the antioxidant capacity was evaluated in terms of DPPH^•^ (18.5 μmol_TroloxEquivalent_.g^−1^ of dry weight (DW)) and ABTS^•+^ (33.7 μmol_TroloxEquivalent_.g_DW_^−1^) assays, with a positive correlation between the carotenoid content and the antioxidant capacity (R^2^ > 0.8).

Furthermore, *Cyanobium* sp. were suggested by Pagels et al. [22] as a source of antioxidant carotenoid-targeted extract. The extract was obtained in an acetonic extraction performed after water extraction, which led to a total carotenoid content of 4 mg.g_DW_^−1^ of extract, with major carotenoids being zeaxanthin and β-carotene. In terms of its antioxidant capacity, the extract was evaluated in ABTS^•+^ and ^•^NO scavenging assays. The IC_50_ were 70 and 162 μg.mL^−1^, respectively.

Another claimed application of carotenoid-targeted extracts from cyanobacteria is about anti-inflammatory capacity. Lopes et al. [21] screened five cyanobacteria including *lkalinema aff. pantanalense*, *Cyanobium gracile*, *Nodosilinea* (*Leptolyngbya*) *antarctica*, *Cuspidothrix issatschenkoi,* and *Leptolyngbya*-like sp. as sources of carotenoid-targeted extracts for the topical treatment of psoriasis. The extracts were obtained using acetone, and the anti-inflammatory capacity against LPS-induced macrophages (RAW 264.7) was evaluated. *Nodosilinea* (*Leptolyngbya*) *antarctica* was the most promising in terms of carotenoids content (64 μg.g_DW_^−1^ of extract, β-carotene and echinenone being the major carotenoids) and in terms of anti-inflammatory capacity, with an IC_50_ of 0.3 mg.mL^−1^.

As cosmeceutical products, carotenoid-targeted extracts from cyanobacteria are associated with anti-ageing agents. Morone et al. [31] compared seven different cyanobacteria strains as hyaluronidase inhibitors, including *Phormidium*, *Synechocystis*, *Nodosilinea*, *Cyanobium,* and *Tychonema* genera. The carotenoids-targeted extracts were obtained using ethanol 70%. *Cyanobium* sp. and *Tychonema* sp. were the ones with the highest inhibition capacity, with IC_50_ of 208 and 182 μg.mL^−1^, respectively. Zeaxanthin and lutein were the main carotenoids identified in *Cyanobium* sp., and canthaxanthin and echinenone were the main carotenoids identified in *Tychonema* sp.

When it comes to the anti-tumoral capacity, Schwartz and Shklar [26] suggested a carotenoid-rich extract of *A. platensis* and *D. salina* (microalgae) as an anti-tumoral agent. The extract was obtained using a mixture of ethyl ether, chloroform, and phosphate buffer saline, 5:1:4. The organic phase was separated and concentrated (1.2 mg.mL^−1^), and the extract contained zeaxanthin (25–30%), myxoxanthophyll (15–20%), β-carotene (10–20%), echinenone (10–15%), and β-cryptoxanthin (5–25%). The extract was applied on 7,12-dimethylbenzanthracene (DMBA)-induced tumour in hamster and led to a local regression of oral squamous cell carcinoma in 4 to 8 weeks. The effect was associated with inhibition of cytokine tumour necrosis factor (TNF-α). Although promising, it is not possible to determine the specific effect of the cyanobacterium and the microalga individually.

Finally, several studies associated the use of *A. platensis* as feed for a higher accumulation of carotenoids in the animals, as in the cases of fish (*Cyprinus carpio*, *Tilapia nilotica*, *T. mossambica*, and *Plecoglossus altivelis*), shrimp (*Penaeus monodon*), and poultry eggs using the raw cyanobacterium as part of the diet (up to 10%) [27,28,29,30].

## 5. Bioprocess Optimization

### 5.1. Production of Carotenoids

Cyanobacteria are found almost everywhere due to the unique mechanism of acclimation and adaptation. They are able to survive in a wide range of conditions, including extreme ones. On the other hand, these different conditions lead to changes within the metabolism and the production of compounds; particularly, the photosynthetic apparatus and its pigments composition may change to maximize survival [33].

When it comes to production for industrial applications, the processing parameters are essential to be optimised to increase production. The growth conditions are also fundamental for the extraction, as they can change the cell structure. Both abiotic (e.g., light, temperature, pH) and biotic factors (e.g., intra- and interspecific competition) must be evaluated, and the optimisation is specific to the species, or even within the strain, although some metabolic responses can be shared between species [4].

In terms of carotenoids, light is the most optimised parameter for the high modulation of photosynthetic metabolism [5]. Light can be optimised in terms of source, quality, intensity, or photoperiod. Apart from that, temperature, pH, and salinity can change the metabolism of the organism in terms of nutrient uptake, growth, and photosynthetic efficiency (consequently pigments composition) [6]. Moreover, the medium composition is also an important factor to be considered, as the concentration of macro- and micronutrients can affect directly the health of the culture and cause unwanted stress [33].

Another way to optimise carotenoids production in cyanobacteria is the use of genetic engineering. In recent years, the approach of genetic alteration increased attention, and a few examples are already described in cyanobacteria. As these organisms produce naturally fewer carotenoids than microalgae, the use of genetic engineering provides an alternative for the competition, although the legislation is yet to restrict the industrial use of genetically modified organisms (GMO) [34].

In the following sections, the main factors and strategies that can affect the production of carotenoids by cyanobacteria are discussed in a way to find similarities between species and provide information for further optimisations.

#### 5.1.1. Light

Photosynthetic organisms depend directly on the availability of light for their growth and survival. Changes in light source, intensity, quality (spectra composition), and photoperiod are responsible for the greatest changes in terms of metabolism, and due to the presence of photoreceptors, the organism can acclimate quickly to the surrounding condition [35]. Optimization of carotenoids production in terms of light is summarized in Table 2.

The photosynthetic process is mainly performed by the light-harvesting complex, composed of pigments (carotenoids, phycobiliproteins, and chlorophylls) [1]. However, light quality can stimulate non-photosynthetic photoreceptors and trigger various pathways, as in the case of carotenoids [36].

Pagels et al. [37] evaluated the pigment accumulation in the cyanobacterium *Cyanobium* sp. using different light qualities supplements. Results showed the positive regulation of carotenoids production under the supplementation of red light, increasing the carotenoid content by 10% when compared to the non-supplemented condition. Afterwards, Pagels et al. [38] evaluated the use of two-phase cultivation using white and red LEDs and, under the red phase, the carotenoids content increased by 50% when compared to the white phase. Moreover, with the optimal cultivation periods of 10 days of white and 4 days of red LEDs, the maximum productivity was of 4.5 mg.L^−1^.d^−1^. The composition of specific carotenoids was not changed within cultivation times, β-carotene being the major carotenoid present.

A similar positive effect of red light was also observed by Olaizola and Duerr [39], who evaluated the potential of *A. platensis* grown under white, blue, and red lights. Red light provided a similar production as white light (23.2 mg.g_DW_^−1^.d^−1^), although the amount of myxoxanthophyll decreased while β-carotene increased in red light, providing a possible strategy for composition modulation.

On the other hand, a negative effect of red light was found in *Pseudanabaena* sp. The comparison between white, green, blue, red, and yellow lights showed that red and yellow induced a decrease in carotenoid content when compared to white light, while green increased the carotenoids content by 30% when compared to white [40]. Such differences are well explained regarding phycobiliproteins, where the mechanisms of acclimation are more known [41]. However, it is possible that a green-red photoreceptor is involved also in carotenoids regulation in cyanobacteria. Such regulation can change within species groups, similarly to what happens in phycobiliproteins regulation.

The mixture of blue and red light is common in plants cultivation and microalgae [42], taking advantage of the chlorophyll absorption peak. However, in cyanobacteria, the absorption of blue light is done in a less effective way [43], and that lack of blue light can reduce carotenoids productivity. Lima et al. [44] evaluated the carotenoids content in *A. platensis* grown in several ratios of red and blue lights. The growth under red:blue (70:30, in percentage) increased up to three times the content of carotenoids (6.91 μg.mL^−1^) when compared to 100% red, while no growth was found under 100% blue.

When it comes to UV radiation, cyanobacteria can produce UV-protective compounds such as phenolic compounds, scytonemins, mycosporine-like amino acids, or carotenoids [45]. Under UV radiation, the cell produces carotenoids due to the NPQ ability of these pigments, reducing oxidative stress and increasing the photosystem stability.

Kokabi et al. [45] saw that *Leptolyngbya* cf. *fragilis* doubles the content of carotenoids within 12 h of exposition to UV radiation (0.29 mg.g_DW_^−1^). A similar pattern was found in *Lyngbya aestuarii*, which progressively increased the carotenoid production with the UV exposure duration. Thus, by 2 days of irradiation treatment, the content of carotenoids increased by 125% when compared to the control [46].

Regarding carotenoid composition, Llewellyn et al. [47] showed that *Chlorogloeopsis fritschii* produced eight times more canthaxanthin under UV radiation when compared to control with a fluorescent lamp, with no differences in other carotenoids content. On the other hand, Ehling-Schulz et al. [48] observed that UV-B irradiation induced an increase in carotenoids, especially echinenone and myxoxanthophyll in *Nostoc commune* after 1 day of irradiation.

In a genetic evaluation, Huang et al. [49] saw an increase in expression of carotenogenesis genes (crtE, crtP, and crtQ) in *Synechocystis* sp. with the addition of high-intensity UV light when compared to white light. Llewellyn et al. [47] also verified that UV radiation induces upregulation of OCP genes expression.

When it comes to light intensity, optimal conditions provide a more efficient photosynthetic metabolism, increasing biomass production and consequently productivity. However, lower and higher amounts of light can induce photoprotective mechanisms, including carotenogenesis. In cyanobacteria, the carotenoids photoprotection effect is described in high light conditions.

Bañares-España et al. [50] evaluated the carotenoids production in three different strains of *Microcystis aeruginosa*, and all of them produced more carotenoids under high light intensity (176 μmol_photons_.m^−2^.s^−1^) when compared to low light intensity (15 μmol_photons_.m^−2^.s^−1^). Thus, Walsh et al. (1997) compared the carotenoids production of *Microcystis aeruginosa* under intensities of 20, 40, and 70 μmol_photons_.m^−2^.s^−1^, 40 μmol_photons_.m^−2^.s^−1^ being the optimal carotenoid production in terms of β-carotene (579.7 μg.g_DW_^−1^), zeaxanthin (431.2 μg.g_DW_^−1^), and echinenone (143.3 μg.g_DW_^−1^), up to three times more than in other intensities.

Moreover, Masamoto and Furukawa [51] compared the accumulation of zeaxanthin in *Synechococcus* sp. grown under 40 and 1300 μmol_photons_.m^−2^.s^−1^ and the results showed that under high light intensity, the cyanobacterium produced 4 times more carotenoids than under low light intensity. A positive correlation between carotenoids production and high intensity was also found in *Anabaena cylindrica*, *Anabaena torulosa*, *Anabaenopsis elenkinii* and *Nostoc* sp. grown under 15 and 120 μmol_photons_.m^−2^.s^−1^ [52].

Cyanobacteria cultures respond to light intensity in a curve response, as it reaches the point of saturation and decreases due to photoinhibition. Pagels et al. [53] showed that *Cyanobium* sp. Increased carotenoids productivity under an optimal light intensity of 200 μmol_photons_.m^−2^.s^−1^, with an increase with the intensity from 50 to 200 μmol_photons_.m^−2^.s^−1^, followed by a decrease at 300 μmol_photons_.m^−2^.s^−1^ with a maximum carotenoid productivity of 0.12 mg.L^−1^.d^−1^. Regarding the carotenoid’s composition, Gris et al. [54] observed that, in *Cyanobacterium aponinum*, zeaxanthin content increased within light intensity from 15 to 650 μmol_photons_.m^−2^.s^−1^, reaching the maximum at 650 μmol_photons_.m^−2^.s^−1^, while β-carotene increased from 15 to 100 μmol_photons_.m^−2^.s^−1^, then deceased until 650 μmol_photons_.m^−2^.s^−1^. Total carotenoids, however, had no significant changes.

**Table 2:** Effects of light quality and intensity on the production of carotenoids by cyanobacteria. Processing parameters include: light source (LS) and intensity (I), light:dark cycle (LC), temperature (T), pH, and culture media (M). Light intensity is expressed in μmol_photons_.m^−2^.s^−1^ unless another unit is indicated.

Cyanobacterium	Tested Conditions ^a^	Processing Parameters ^b^	Optimal Condition	Carotenoids Content	Reference
**Light Quality**					
*Arthrospira platensis*	W, B, R	**LS**: FL; **I**: 133; **LC**: 24:0 h; **T**: 36 °C; **pH**: ns; **M**: Zarrouk	R	23.2 mg.g_DW_^−1^	[39]
*Arthrospira platensis*	R + B (0–100% mixtures)	**LS**: LED; **I**: 100; **LC**: 24:0 h; **T**: 32 °C; **pH**: 8.0; **M**: Zarrouk	R + B (70:30, %)	6.91 μg.mL^−1^	[44]
*Cyanobium* sp.	R; G; B; UV supplements	**LS**: SOX + LED; **I**: 200; **LC**: 12:12 h; **T**: 25 °C; **pH**: 7.5; **M**: BG11 saline	R supplement	6.5 mg.g_DW_^−1^	[37]
*Cyanobium* sp.	W + R in different times	**LS**: LED; **I**: 200; **LC**: 16:8 h; **T**: 20 °C; **pH**: 9; **M**: BG11 saline	10 days of W and 4 days of R	32 mg.g_DW_^−1^	[38]
*Leptolyngbya* cf. *fragilis*	(+/−) UV-B	**LS**: FL + UV lamp; **I**: 18 W.m^−2^; **LC**: 12:12 h; **T**: 25 °C; **pH**: ns; **M**: BG11	+UV	0.29 mg.g_DW_^−1^	[45]
*Lyngbya aestuarii*	(+/−) UV-B	**LS**: FL + UV lamp; **I**: 7.5 W.m^−2^ FL or 5 W.m^−2^ UV; **LC**: 24:0 h; **T**: 25 °C; **pH**: ns; **M**: ASN-III	+UV	ns	[46]
*Nostoc commune*	(+/−) UV-B	**LS**: FL + UV lamp; **I**: 1 W.m^−2^; **LC**: ns; **T**: 30 °C; **pH**: ns; **M**: BG11_0_	+UV-B	ns	[48]
*Pseudanabaena* sp.	W, B, R, G, Y	**LS**: FL + colour filters; **I**: 75–220 lux; **LC**: 12:12 h; **T**: 25 °C; **pH**: ns; **M**: ASN-III	G	0.16 mg.L^−1^	[40]
*Synechocystis* sp.	(+/−) UV-B	**LS**: FL + UV lamp; **I**: 60; **LC**: ns; **T**: 30 °C; **pH**: ns; **M**: BG11	+UV	ns	[49]
**Light Intensity**					
*Cyanobium* sp.	50, 100, 200, 300	**LS**: FL or SOX; **LC**: 12:12 h; **T**: 25 °C; **pH**: 7.5; **M**: BG11 saline	200	0.12 mg.L^−1^.d^−1^	[53]
*Synechococcus* sp.	40, 1300	**LS**: FL; **LC**: 24:0 h; **T**: 25 °C; **pH**: ns; **M**: BG11	1300	7.59 nmol.A_750_^−1^.mL^−1^	[51]
*Microcystis aeruginosa*	20, 40, 70	**LS**: FL; **LC**: 12:12 h; **T**: 25 °C; **pH**: ns; **M**: ASM	40	β-carotene: 579.7 μg.g_DW_^-1^	[55]
				Zeaxanthin: 431.2 μg.g_DW_^-1^	
				Echinenone: 143.3 μg.g_DW_^-1^	
*Anabaena cylindrica*	15, 120	**LS**: FL; **LC**: 24:0 h; **T**: 20 °C; **pH**: ns; **M**: Juttner	120	ns	[52]
*Anabaenopsis elenkinii*	15, 120	**LS**: FL; **LC**: 24:0 h; **T**: 20 °C; **pH**: ns; **M**: Juttner	120	ns	[52]
*Anabaena torulosa*	15, 120	**LS**: FL; **LC**: 24:0 h; **T**: 20 °C; **pH**: ns; **M**: Juttner	120	ns	[52]
*Nostoc* sp.	15, 120	**LS**: FL; **LC**: 24:0 h; **T**: 20 °C; **pH**: ns; **M**: Juttner	120	ns	[52]
*Microcystis aeruginosa*	15, 176	**LS**: FL; **LC**: 16:8 h; **T**: 20 °C; **pH**: ns; **M**: BG11	176	ca. 0.035 pg.cell^−1^	[50]
*Cyanobacterium aponinum*	15, 40, 70, 100, 150, 300, 500, 650	**LS**: FL; **LC**: 24:0 h; **T**: 35 °C; **pH**: 8.0; **M**: BG11	β-carotene: 100	β-carotene:4.03 mg.g_DW_^−1^	[54]
			Zeaxanthin: 650	Zeaxanthin: 3.17 mg.g_DW_^−1^	

^a^ Light quality: R—red; G—green; B—blue; UV—ultraviolet; Y—yellow; W—white; ^b^ SOX—low-pressure sodium lamp; LED—light emitting diodes; FL—fluorescent lamp; ns–not specified.

#### 5.1.2. Temperature and pH

Both temperature and pH exert a great influence on cyanobacterial metabolism due to nutrient uptake and solubility of CO_2_ in the culture medium. These factors can also change enzymatic activity and consequently metabolic pathways of the organism [56]. In the case of cyanobacteria, a wide range of values of the two factors is tolerable, and these organisms are found even in the most extreme environments, such as hot springs, Antarctica, or even saline-alkaline lakes [57,58]. Most of the studies regarding both temperature and pH in cyanobacteria production are related to biomass or phycobiliproteins, and only a few have targeted carotenoids production, summarized in Table 3.

Kłodawska et al. [59] evaluated the effect of temperature (15, 23, 30, and 37 °C) in carotenoids production in *Anabaena* sp., and results showed that optimal temperature for carotenoids production was 23 °C (0.39 mg.g_DW_^−1^), although the ratio carotenoids:chlorophyll did not change between 23 °C and 30 °C. However, in terms of composition of β-carotene and echinenone, the optimal temperature was 23 °C, while for keto-myxoxanthophyll and canthaxanthin, the optimal temperature was 30 °C.

Ismaiel et al. [60] evaluated the effect of pH (7.5–11.0) in carotenoids production in *A. platensis*, and the results showed that the content of carotenoids was higher in pH from 8.0 to 9.0 with no statistical differences (ca. 2.4 mg.g_DW_^−1^).

Pagels et al. [61] performed a factorial evaluation of temperature (20–30 °C) and pH (6.0–9.0), together with salinity (see Section 5.1.4) on *Cyanobium* sp. for the production of carotenoids. Optimal conditions were set at 20 °C and pH 9.0, with a maximum productivity of 2.04 mg.L^−1^.d^−1^. It is noteworthy that the carotenoid content in the optimal condition for productivity is not the maximum content on the cyanobacterium, however, the amount of the final product can have more impact on the decision-making than the biomass composition.

Overall, more studies are required for a better characterization of temperature and pH needs of cyanobacterial cultures as a source of carotenoids.

**Table 3:** Effects of temperature and pH on the production of carotenoids by cyanobacteria. Processing parameters include: light source (LS) and intensity (I), light:dark cycle (LC), temperature (T), pH, and culture media (M). Light intensity is expressed in μmol_photons_.m^−2^.s^−1^ unless another unit is indicated.

Cyanobacterium	Tested Conditions	Processing Parameters ^a^	Optimal Condition	Carotenoids Content	Reference
**pH**					
*Arthrospira platensis*	7.5–11.0	**LS**: FL; **I**: 60; **LC**: ns; **T**: 31 °C; **M**: Zarrouk	8.0-9.0	2.4 mg.g_DW_^−1^	[60]
*Cyanobium* sp.	6.0–9.0	**LS**: FL; **I**: 200; **LC**: 16:8 h; **T**: 20 °C; **M**: BG11 saline	9.0	2.04 mg.L^−1^.d^−1^	[61]
**Temperature**					
*Anabaena* sp.	15, 23, 30, 37 °C	**LS**: FL; **I**: 60; **LC**: 24:0 h; **pH**: 7.5; **M**: BG11_0_	23 °C	0.39 mg.g_DW_^−1^	[59]
*Cyanobium* sp.	20–30 °C	**LS**: FL; **I**: 200; **LC**: 16:8; **pH**: 9.0; **M**: BG11 saline	20 °C	2.04 mg.L^−1^.d^−1^	[61]

^a^ FL–fluorescent lamp.

#### 5.1.3. Culture Medium Composition

Another important factor to be optimised is the appropriate chemical composition of the growth media. Nitrogen, phosphorous, sulphur, magnesium, and manganese are the most essential nutrients for both growth and carotenoids accumulation. The nutritional needs of cyanobacteria species require case-wise consideration in terms of nutrients concentration or source of nitrogen. Several optimised media are used by laboratory and industrial productions of cyanobacteria, such as Zarrouk and Blue-Green (BG11) media. Table 4 summarizes the optimisation of medium components in cyanobacteria production as a carotenoids source.

Regarding nutrients concentration, Thirumala [62] optimised the medium nutrients to *A. platensis* isolated from Lonar Lake, Mexico. The optimisation used sterilized water from the lake in addition to a mixture of N:P:K (1:1:1) (0–2.5 g.L^−1^), with an optimal concentration of 2 g.L^−1^ and with a carotenoid’s concentration of 0.0998 μg.mL^−1^, 14% higher than compared to Zarrouk medium. In another study, *A. platensis* was optimised in terms of NaNO_3_ concentration (0.1, 2.5, and 5.0 g.L^−1^), and the optimal condition (0.1 g.L^−1^) led to a total of 45.4 mg.g^−1^ of carotenoids, 60% more than the culture with 2.5 g.L^−1^ and 750% more than the one with 5.0 g.L^−1^ [63]. Moreover, Pagels et al. [53] suggested the addition of twice as many nitrates and phosphates than regular BG11 medium suggests for the production of carotenoids by *Cyanobium* sp., and the addition of nutrients induced an increase of 20% in productivity.

When it comes to the source of nitrogen, Erdoğan et al. [64] evaluated the sources of nitrogen (NaNO_3_, NaNO_2_, NH_4_Cl, and CH_4_N_2_O) in *Prochlorococcus* sp. culture for the production of lutein. The maximum concentration of lutein (3.34 mg.g_DW_^−1^) was found in the culture grown with CH_4_N_2_O.

Another optimisation strategy is to use different laboratory medium. Tarko et al. [65] evaluated the β-carotene content in six strains of *A. platensis* grown with Zarrouk and Revised No. 6 (RM6) media. In all strains, β-carotene was more produced in Zarrouk medium, with a higher concentration (2.26 mg.g_DW_^−1^) 15 times more than RM6 medium.

Moreover, a comparison between Chu’s No. 10 (CHU10), BG11, and Zarrouk media was performed by Paliwal et al. (2015b) for the production of *Synechocystis* sp., Zarrouk being the optimal medium with a content of 7.99 mg.g_DW_^−1^, leading to content 50% higher than BG11 and 80% higher than CHU10. Furthermore, D’Alessandro et al. [66] compared the carotenoids´ production by *Geitlerinema amphibium* using Wright’s Cryptophyte (WC) and Bold’s Basal (BBM) media. The culture grown using BBM medium produced 130% more astaxanthin and 234% more lutein than the one grown using WC medium, 2.74 mg.g_DW_^−1^ and 5.49 mg.g_DW_^−1^ respectively.

**Table 4:** Effects of culture medium composition on the production of carotenoids by cyanobacteria. Processing parameters include: light source (LS) and intensity (I), light:dark cycle (LC), temperature (T), pH, and culture media (M). Light intensity is expressed in μmol_photons_.m^−2^.s^−1^ unless another unit is indicated.

Cyanobacterium	Tested Culture Media	Processing Parameters ^a^	Optimal Condition	Carotenoids Content	Reference
**Culture Media Comparison**					
*Arthrospira platensis*	Zarrouk and RM6	**LS**: ns; **I**: 2000–3000 lux; **LC**: 12:12 h; **T**: 20 °C; **pH**: 8.2;	Zarrouk	2.26 mg.g_DW_^−1^	[65]
*Synechocystis* sp.	CHU10, GB11 and Zarrouk	**LS**: ns; **I**: 60; **LC**: ns; **T**: 25 °C; **pH**: ns;	Zarrouk	7.99 mg.g_DW_^−1^	[67]
*Geitlerinema amphibium*	WC and BBM	**LS**: ns; **I**: 80; **LC**: 24:0h; **T**: 29 °C; **pH**: ns;	BBM	Astaxanthin: 2.74 mg.g_DW_^−1^ Lutein: 5.49 mg.g_DW_^−1^	[66]
**Nutrient’s concentration**					
*Arthrospira platensis*	0–2.5 g.L^−1^ of N:P:K (1:1:1)	**LS**: ns; **I**: 1500 lux; **LC**: 14:10 h; **T**: 35 °C; **pH**: 10; **M**: Lonar lake water	2 g.L^−1^	0.0998 μg.mL^−1^	[62]
*Arthrospira platensis*	0.1–5 g.L^−1^ of NaNO_3_	**LS**: FL; **I**: 475 lux; **LC**: 24:0 h; **T**: 25 °C; **pH**: 9.5; **M**: Zarrouk	0.1 g.L^−1^	45.54 mg.g_DW_^−1^	[63]
*Cyanobium* sp.	(+/−) NaNO_3_; K_2_HPO_4_	**LS**: SOX; **I**: 200; **LC**: 12:12 h; **T**: 25 °C; **pH**: 7.5; **M**: BG11 saline	+NaNO_3_; K_2_HPO_4_	0.12 mg.L^−1^.d^−1^	[53]
**Nitrogen source**					
*Prochlorococcus* sp.	NaNO_3_, NaNO_2_, NH_4_Cl, CH_4_N_2_O	**LS**: FL; **I**: 27; **LC**: 24:0 h; **T**: 25 °C; **pH**: ns; **M**: BBM	CH_4_N_2_O	3.34 mg.g_DW_^−1^	[64]

^a^ SOX—low-pressure sodium lamp; FL—fluorescent lamp; ns—not specified.

#### 5.1.4. Salinity

When dealing with marine cyanobacteria, salinity is a key point for optimisation. Higher concentrations of salt (NaCl ≈ 30 g.L^−1^), means the use of less potable water, the seawater being a suitable substitute for cultivation. However, the salt concentration in the culture medium influences not only growth and carotenoids accumulation but can also influence biomass harvesting and carotenoids extraction. Table 5 summarizes the optimisation of salinity in cyanobacteria production as a carotenoids source.

Pagels et al. [61] optimised salinity in terms of NaCl concentration (10 to 30 g.L^−1^), together with temperature and pH (see Section 5.1.2). Optimal concentration was set at 10 g.L^−1^ (NaCl), with a carotenoid productivity of 2.04 mg.L^−1^.d^−1^. From the three studied factors, NaCl concentration had the least impact in terms of carotenoids production.

In the case of *Euhalothece* sp., a halophilic cyanobacterium that is capable of growing in a wide range of salinity, from freshwater to 70 g.L^−1^ of NaCl, the carotenoid content was 300% higher in the condition without NaCl addition than the condition with 30 g.L^−1^. In contrast, the growth was reduced by 400%, meaning that the salt stress-induced a defence mechanism, increasing the carotenoid content but inhibiting biomass production [68].

**Table 5:** Effects of salinity on the production of carotenoids by cyanobacteria. Processing parameters include: light source (LS) and intensity (I), light:dark cycle (LC), (T), pH, and culture media (M). Light intensity is expressed in μmol_photons_.m^−2^.s^−1^ unless when another unit is indicated.

Cyanobacterium	Tested [NaCl]	Processing Parameters ^a^	Optimal Condition	Carotenoids Content	Reference
*Cyanobium* sp.	10–30 g.L^−1^	**LS**: FL; **I**: 200; **LC**: 16:8; **T**: 20 °C; **pH**: 9.0; **M**: BG11 saline	10 g.L^−1^	2.04 mg.L^−1^.d^−1^	[61]
*Euhalothece* sp.	0 and 30 g.L^−1^	**LS**: ns; **I**: 75; **LC**: 14:10; **T**: 27 °C; **pH**: 7.5; **M**: BG11	0 g.L^−1^	0.61 μg.A_750_^−1^	[68]

^a^ FL—fluorescent lamp; ns—not specified.

#### 5.1.5. Genetic Engineering

As already referenced, compared to microalgae, cyanobacteria content of carotenoid is generally low and thus has difficulty competing in the market. Another strategy for the increase of the production of carotenoids from cyanobacteria is the use of genetic engineering.

*Synechocystis* sp. PCC 6803 is the most studied cyanobacteria in terms of genetic engineering. In the case of carotenoids production, Lagarde et al. [69] overexpressed carotenogenesis genes in *Synechocystis* sp. by introducing sequences of the genes encoding the yeast isopentenyl diphosphate isomerase (ipi) and the *Synechocystis* β-carotene hydroxylase (crtR) as well as the linked *Synechocystis* genes coding for phytoene desaturase and phytoene synthase (crtP and crtB, respectively). The gene introduction led to an overexpression of crtP and crtB, increasing the production of myxoxanthophyll and zeaxanthin by 50%. On the other hand, the overexpression of crtR increased the production of zeaxanthin by 150% (from 0.39 to 0.98 mg.L^−1^.OD_730_^−1^), but it also led to a reduction of echinenone and β-carotene by 50%.

Moreover, Diao et al. [13] induced astaxanthin biosynthesis through genetic engineering in *Synechocystis* sp. PCC 6803, reaching a production of 29.6 mg.g_DW_^−1^, representing a 500-fold increase when compared to the wild type strain. Astaxanthin biosynthesis is established in *Synechocystis* by introducing two carotenogenesis enzymes: β-carotenoid ketolase and hydroxylase. The source of the genes and the expression of the chassis determine the efficiency of the process and the astaxanthin content.

Furthermore, Gao et al. [70] overexpressed the crtO gene from *Nostoc flagelliforme* into *Nostoc* sp. PCC 7120, inducing production of 16% more echinenone (97.9 mg.L^−1^) and 80% more canthaxanthin (8.8 mg.L^−1^).

Genetic engineering of cyanobacteria was also demonstrated to be a viable option for cyanobacteria-derived terpenoids, such as GGPP, a precursor to carotenoids (Section 2). GGPP commercial application also includes pharmaceuticals, nutraceuticals, flavours/fragrances, and industrial chemicals. Terpene production, on the other hand, would inevitably compete with pigment synthesis due to the use of the same precursor pathway, decreasing the final content of carotenoids [71].

### 5.2. Downstream Process

Downstream processing involves harvesting, extraction, and purification processes. In terms of harvesting, the critical optimisation step is about feasibility on a large scale and its cost, because, generally, the costs of harvesting represent about 20–30% of the total cost of biomass production. Filtration, centrifugation, or even chemical flocculation are some of the options available, but the process must be optimised for individual species and purposes. Optimization of harvesting is targeted to the whole biomass; this review does not cover these aspects, but more aspects of harvesting optimisation for cyanobacteria can be found in Guedes et al. [33]. Moreover, carotenoids are usually commercialized in a form of extract (oleoresin) containing a high concentration of specific carotenoids (e.g., β-carotene representing about 20% of the extract). Further purification can be performed, but the cost is hardly justifiable [72].

In terms of extraction, many factors can change the quality of the final product, including target carotenoid, chosen cyanobacteria, available technology, and cost. Most cyanobacteria biomass requires cell disruption associated with solvent extraction, and, in general, extraction of carotenoids is performed by physical disruption of cells (such as pressurized, wave-based, or electric fields technologies). Associated with this is the choice of a compatible organic solvent, preferentially considered safe for industrial use (GRAS solvents), as in the cases of acetone, ethanol, or hexane [22]. The extraction optimisation must consider, if possible, both disruption methodology and compatible solvent (with the system and with the targeted compound). Moreover, is it important to control the temperature through the extraction process due to the thermosensitivity of carotenoids. Carotenoids can be more efficiently extracted under 50 °C to 65 °C, but long expositions to high temperatures (>70 °C) can lead to degradation and loss of the bioactive capacity of the extract [73,74].

Thus, the first step of extraction optimisation must be in terms of solvents. Carotenoids are lipophilic compounds and must be extracted using organic solvents. Table 6 summarizes the solvent optimisations performed in carotenoids extraction from cyanobacteria. Amaro et al. [75] evaluated the effect of different solvents in the carotenoid’s extraction in *Gloeothece* sp. and compared the use of ethanol, acetone, ethyl lactate, and a mixture of hexane/isopropanol (60:40, in percentages). The results showed that acetone was the best solvent, with a yield of 1.8 mg.g_DW_^−1^, with lutein being the major carotenoid (ca. 80% of total carotenoids), followed by β-carotene, neoxanthin, violaxanthin, and α-carotene. The use of acetone was 40% more efficient than ethanol and hexane/isopropanol (60:40, in percentages) mixture. Noteworthy is that ethyl lactate extracted 70% fewer carotenoids, and α-carotene was not found in this extract.

Another strategy for solvent extraction involves the use of more than one solvent in a successive way, e.g., successive extractions using the remaining biomass from the previous process. This strategy is gaining attention in the last few years and can contribute to the valorisation of carotenoids from cyanobacteria using also the biomass for the extraction of aqueous pigments phycobiliproteins, with high added value in the market [15]. Moreover, the use of successive extraction can increase the purity of both phycobiliproteins and carotenoids extracts, as they are separated into two different extracts.

Tavanandi et al. [76] optimised the extraction of carotenoids from *Arthrospira platensis* after an enzymatic extraction of phycobiliproteins. The first extraction was performed with lysozyme for 20 h at 37 °C and pH 7.0. The remaining biomass was then dried by low humidity drying. Then, the carotenoids extraction was optimised comparing ethanol, acetone, methanol, diethyl ether and DMSO; pH (4.0–10.0); and time of extraction (1–14 h). The optimal condition was ethanol (80%), pH 7.0, stirring for 1 h at 40 °C, with a content of 5.3 mg.g_DW_^−1^.

Pagels et al. [22] optimised the successive extraction of pigments from *Cyanobium* sp., comparing the solvent (acetone, ethyl acetate, and ethanol) and if the extraction should be before the aqueous extraction or after (acetone after water extraction). In terms of the composition of the extract, acetone as the first or the second extraction reached the highest concentration of carotenoids in the extract (ca. 4 mg.g_DW_^−1^), β-carotene being the major carotenoid, followed by echinenone, zeaxanthin, and lutein.

The successive extraction of carotenoids was also evaluated in *Trichocoleus sociatus* and *Nostoc flagelliforme* by Dorina et al. [77]. The optimisation considered the order of extracted compounds—carotenoids plus chlorophylls, phycobiliproteins, and exopolysaccharides. The optimal condition was found in the extraction of exopolysaccharides prior to biomass drying, followed by a freeze-drying step, an aqueous extraction for phycobiliproteins, and finally a methanol extraction for the obtention of carotenoids, with yields of 2.34 mg.g_DW_^−1^ for *Trichocoleus sociatus* and 4.49 mg.g_DW_^−1^ for *Nostoc flagelliforme*.

Finally, Assunção et al. [78] evaluated the successive extraction in *Chroococcidiopsis* sp. In this study, acetone, ethanol, and methanol were used for the extraction of carotenoids after a pre-treatment with phosphate buffer, where the extraction with methanol led to the highest content of these pigments (1.72 mg.g_DW_^−1^), mainly composed of echinenone, β-carotene, α-carotene, lycopene, and zeaxanthin.

**Table 6:** Optimisation of solvent in the successive extraction of carotenoids from cyanobacteria.

Cyanobacterium	Tested Solvents	Optimal Solvent	Carotenoid Content (mg.g_DW_^−1^)	Main Identified Carotenoids	Reference
*Gloeothece* sp.	Ethanol, acetone, ethyl lactate, and hexane/isopropanol (60:40, in percentages).	Acetone	1.8	Lutein, β-carotene, neoxanthin, violaxanthin, and α-carotene	[75]
*Arthrospira platensis*	Ethanol, acetone, methanol, diethyl ether, and DMSO after enzymatic pre-treatment	Ethanol	5.3	n.s.	[76]
*Cyanobium* sp.	Acetone, ethyl acetate, and ethanol	Acetone	4.4	β-carotene, echinenone, zeaxanthin, and lutein	[22]
*Chroococcidiopsis* sp.	Acetone, ethanol, and methanol	Methanol	1.7	Echinenone, β-carotene, α-carotene, lycopene, and zeaxanthin	[78]

n.s.—not specified.

For a better extraction efficiency, the cell disruption methodology must be chosen in terms of the specific cell wall composition and the scalability of the technology. Unlike other Gram-negative bacteria and microalgae, cyanobacteria contain a thick peptidoglycan layer between the inner and the outer membrane, which can increase the resistance of the cell in the extraction process [79]. Regarding cyanobacteria, Table 7 summarizes the main technologies used for carotenoids extraction.

In the case of pressurized liquid extraction (PLE), the extraction is performed at high temperatures (50–200 °C) and high pressure (100–140 bar), preventing the solvent from boiling while increasing the solvent flux into the cell. The major limitation is that temperature can induce degradation of pigments [80]. Rodríguez-Meizoso et al. [81] optimised carotenoid extraction from *Phormidium* spp. in terms of solvent (hexane and ethanol) and temperature (50–200 °C) in a constant pressure (100 bar). The optimal condition was ethanol at 150 °C, the major carotenoid being β-carotene, followed by lutein, violaxanthin, and neoxanthin. Moreover, Amaro et al. [82] used a low temperature pressurized liquid extraction for carotenoids obtention from *Gloeothece* sp. The optimisation was performed using ethanol in terms of biomass in the system (50–150 mg_DW_), flow (1–4 mL.min^−1^), temperature (30–70 °C), and cycles of solvent recirculation. The optimal condition was set as 50 mg_DW_, 60 °C, a flow of 3 mL.min^−1^ (180 bar), and three cycles of ethanol recirculation, with contents of 2.9 mg.g_DW_^−1^ of lutein and 1.5 mg.g_DW_^−1^ of β-carotene.

In terms of ultrasound-assisted extraction (UAE), the extraction uses acoustic cavitation for cell disruption. In cyanobacteria, the use of UAE was performed for carotenoid extraction in *A. platensis* [83]. The optimisation was performed in terms of solvent (hexane, n-heptane and diethyl ether), temperature (10–50 °C) and electrical acoustic intensity (64–210 W.cm^−2^). Optimal conditions were found by using heptane at a temperature of 30 °C and an electrical acoustic intensity of 167 W.cm^−2^, with content of ca. 1.0 mg.g_DW_^−1^.

Furthermore, supercritical fluid extraction (SFE) can be an efficient but expensive extraction method for the obtention of carotenoids. In supercritical condition, the solvent acts as gas and liquid at the same time, penetrating the cell and solubilizing the carotenoids. In cyanobacteria, Montero et al. [84] optimised carotenoid extraction in *Synechococcus* sp. in terms of pressure (200–500 bar) and temperature (40–60 °C). Optimal conditions were carotenoid-specific: 358 bar and 50 °C for β-carotene; 454 bar and 59 °C for cryptoxanthin; and 500 bar and 60 °C for zeaxanthin.

Similarly, Macías-Sánchez et al. [85] optimised carotenoids extraction in *Synechococcus* sp. using SFE. The optimal pressure was set at 300 bar (from 100 to 500 bar) and a temperature of 50 °C (from 40 to 60 °C), reaching a content of 1.5 mg.g_DW_^−1^.

Other cell disruption methodologies are associated with the extraction of carotenoids in microalgae, and some could be easily applied in cyanobacteria matrices, as in the case of enzymatic extraction [86], high-pressure homogenization [87], microwave-assisted extraction [88], or electroextraction [89]. Moreover, from the described for cyanobacteria, the extraction efficiency of supercritical fluid extraction can be increased by the use of co-solvents, such as ethanol, that increases the solvating power, as observed in *Scenedesmus obliquus* lutein extraction [90].

**Table 7:** Extraction methodologies used for the obtention of carotenoids from cyanobacteria.

Cyanobacterium	Extraction Method	Carotenoid Content (mg.g_DW_^−1^)	Main Identified Carotenoids	Reference
*Phormidium* spp.	Pressurized liquid extraction	n.s.	β-carotene, followed by lutein, violaxanthin, and neoxanthin	[81]
*Gloeothece* sp.	Continuous pressurized solvent extraction	Lutein: 2.9 β-carotene: 1.5	Lutein, β-carotene, neoxanthin, violaxanthin and α-carotene	[82]
*Arthrospira platensis*	Ultrasound-assisted extraction	ca. 1.0	β-carotene	[83]
*Synechococcus* sp.	Supercritical fluid extraction	ca. 2.0	β-carotene, zeaxanthin, myxoxanthophyll and β-cryptoxanthin	[84]
*Synechococcus* sp.	Supercritical fluid extraction	1.5	n.s.	[85]

n.s.—not specified.

## 6. Economical Perspective

The market for natural pigments is increasing due to an urge to replace synthetic pigments with natural and sustainable sources. This demand is raising as the European Union drives the use of microalgae as a source of carotenoids. As the main consumer of carotenoids (followed by North America and Asia), Europe plays a key role in the carotenoids market [8].

In terms of application, food is the main industrial sector for carotenoids. This market alone is expected to reach over $2 billion (USD) worldwide in 2026 [8]. Moreover, with the development of fundamental research, carotenoids are being seen as bioactive compounds with high potential to improve human health.

From microalgae, two successful cases of carotenoids bioprocess are well described: astaxanthin from *H. pluvialis* and β-carotene from *D. salina*. From cyanobacteria, the only successful pigment production is related to phycocyanin from *A. platensis*, with no market application for carotenoids from cyanobacteria [6].

By evaluating all the details through this review, a question is yet to be answered: is it economically feasible to produce carotenoids from cyanobacteria? The answer is not exactly clear and not favourable to cyanobacteria. The content of carotenoids in cyanobacteria is extremely low when compared to microalgae such as *D. salina*, which has a β-carotene content of up to 14% of dry weight. In this review, the highest content of carotenoid was found in *A. platensis* (ca. 4% of dry weight) [63]. However, considering that the phycobiliproteins content in *A. platensis* is up to 20% of dry weight [91], it would be possible, if co-produced, to recover about 24% of dry weight in pigments in two different products.

Mitra and Mishra [92] suggested a biorefinery process using *A. platensis* for the obtention of phycocyanin, β-carotene, polyhydroxyalkanoates (PHA), and biofuel. *A. platensis* biomass production represents more than half of total microalgae and cyanobacteria production, estimating a total market of 780 million (USD) by 2026 [92]. In an optimal scenario, it is possible to use the biomass for this variety of products through a process of several extractions to increase the economic feasibility of cyanobacteria-based bioprocess [93]. Moreover, the reduction of biomass waste and the valorisation of the whole bioprocess makes the exploitation of cyanobacteria a more sustainable and environmentally friendly approach than their market competitor (microalgae).

## 7. Final Remarks

Carotenoids have been an interest of science and industry for several years. More recently, with increasing demand, new sources are being evaluated, including cyanobacteria, although in a less pronounced way when compared to microalgae or vascular plants. Moreover, the bioactive potential and the application of these carotenoids or carotenoid-rich extracts are promising but are still in a general perspective of screenings and are not conclusive or applied.

When it comes to production, the optimisation of processing parameters was targeted by fundamental and applied research, and great development can be done in the next years if aiming for, for example, a scaling-up process. A similar strategy might be seen in extraction processes as well. Moreover, the development of a biorefinery process of co-production of pigments or even other compounds should be proposed for better use of the biomass and a more sustainable bioprocess.

Overall, cyanobacteria are already seen as potential candidates for numerous applications, and their carotenoids must be considered as a product to be explored. Thus, it is expected that there are other uses, and more studies should be done about these compounds in these organisms.

## Data Availability

Not applicable.
